# Expertise in action observation: recent neuroimaging findings and future perspectives

**DOI:** 10.3389/fnhum.2013.00637

**Published:** 2013-10-16

**Authors:** Luca Turella, Moritz F. Wurm, Raffaele Tucciarelli, Angelika Lingnau

**Affiliations:** ^1^Center for Mind/Brain Sciences (CIMeC), University of TrentoTrento, Italy; ^2^Department of Cognitive Sciences, University of TrentoTrento, Italy

**Keywords:** fMRI, expertise, action observation network, mentalizing system

## Introduction

In everyday life, we continuously interact with other individuals. Understanding actions of other people, i.e., the ability to distinguish between different actions, such as passing over vs. threatening someone with a knife, has been crucial for the survival of our species and is a fundamental capability for our social interactions.

**Figure 1:**
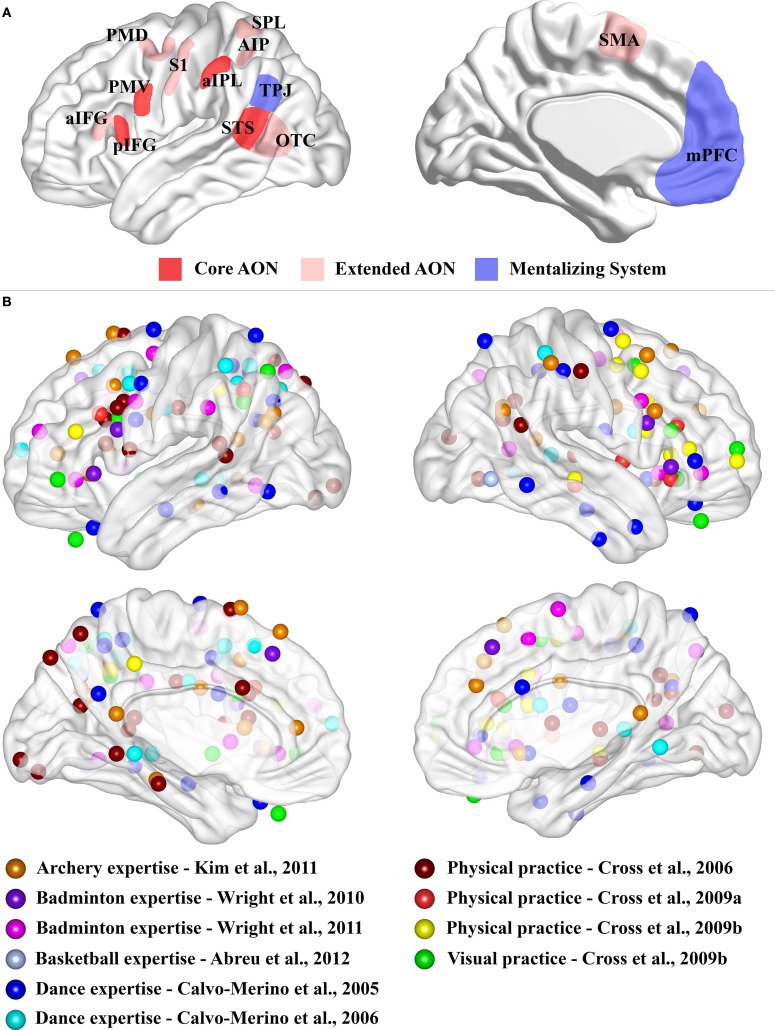
**(A)** Schematic representation of the core AON, extended AON and mentalizing system. Three-dimensional representation of lateral and medial brain surface. The regions assigned to the “core” AON, “extended” AON and the “mentalizing” system are depicted. In red, the core AON is presented comprising: the PMV/pIFG complex, the aIPL and the STS. In pale red, the “extended” AON is presented comprising: the anterior part of the inferior frontal gyrus, (aIFG), the dorsal premotor cortex (PMD), the supplementary motor area (SMA), the superior parietal lobule (SPL), the anterior intraparietal sulcus (AIP), the somatosensory cortex (S1) and the occipito-temporal cortex (OTC), including also STS. The mentalizing system (blue) is assumed to consist of the medial prefrontal cortex (mPFC) and the temporo-parietal junction (TPJ). Note that the extension of these networks is not representative of their real dimension or functional significance. **(B)** Expertise effects. Three-dimensional representation of lateral and medial brain surface with location of peaks for the comparisons of interest superimposed. For Kim et al. (2011), we considered the comparison between the two groups (Table 2). For Wright et al. (2010), we considered results from ROI analysis (Table 2). For Wright et al. (2011), we plotted results for normal video (Table 2). For Abreu et al. (2012), we used the peak of the significant cluster within the temporal lobe in the group comparison (page 1649 of the manuscript). For Calvo-Merino et al. (2005), we plotted the reported interaction (see Table 1). For Calvo-Merino et al. (2006), we plotted the results from Table S2. For Cross et al. (2006), we considered the main effect of the contrast of interest (Table 2). For Cross et al. (2009a), we reported the contrast for physical training (Danced > Untrained) (Table 1). For Cross et al. (2009b), we considered the physical training results (Table 1) and the observational training results (Table 1). We excluded the peaks located within the cerebellum.

Neuroimaging studies investigated the neural substrates subtending action perception using a variety of techniques, ranging from univariate analysis of fMRI data (Brass et al., 2007; Gazzola et al., 2007; De Lange et al., 2008; Gazzola and Keysers, 2009; Turella et al., 2009a, 2012; Wurm et al., 2011; Wurm and Schubotz, 2012; Wurm et al., 2012; Lingnau and Petris, 2013), to fMRI repetition suppression (Dinstein et al., 2007; Chong et al., 2008; Lingnau et al., 2009; Kilner et al., 2009) and multivoxel pattern analysis (MVPA; Dinstein et al., 2008a; Oosterhof et al., 2010, 2012). These studies reported the consistent recruitment of a number of regions, generally assumed as pertaining to two different networks, typically referred to as the action observation network (AON) and the mentalizing system (Figure 1A). Both networks have been advocated to be involved in action understanding (Brass et al., 2007; De Lange et al., 2008; Van Overwalle, 2009; Van Overwalle and Baetens, 2009; Wurm et al., 2011), but their precise roles and their causal involvement are strongly debated (Dinstein et al., 2008b; Mahon and Caramazza, 2008; Hickok, 2009; Turella et al., 2009b; Rizzolatti and Sinigaglia, 2010).

In homology with monkey neurophysiological studies, three regions have been proposed to form the human AON (Rizzolatti and Craighero, 2004; Rizzolatti and Sinigaglia, 2010; see Figure 1A). This “core” AON was defined as comprising (i) the ventral premotor cortex (PMV) together with the posterior part of the inferior frontal gyrus (pIFG), (ii) the anterior inferior parietal lobule (aIPL) and (iii) the superior temporal sulcus (STS). Human neuroimaging studies suggested the recruitment of several additional areas that were incorporated in an “extended” version of the AON (Gazzola and Keysers, 2009; Caspers et al., 2010; see Figure 1A).

The *mentalizing system* has been identified in human neuroimaging studies investigating social cognition tasks, such as intention and beliefs attribution about the self or others, while observing action-related stimuli (Van Overwalle, 2009). The regions consistently assigned to this network are the medial prefrontal cortex (mPFC) and the temporo-parietal junction (TPJ) (Figure 1A), and less often also the precuneus and the posterior cingulate cortex (Amodio and Frith, 2006; Brass et al., 2007; De Lange et al., 2008; Van Overwalle, 2009; Van Overwalle and Baetens, 2009).

The first description of the involvement of sensorimotor regions during action perception started with the discovery of mirror neurons in the ventral premotor cortex in macaque monkeys (Di Pellegrino et al., 1992). These visuomotor neurons responded both while the monkey executed or observed similar actions and were later described also within the monkey inferior parietal lobule (Fogassi et al., 2005). Note that both regions also contain neurons with motor-only and visual-only properties (Gallese et al., 1996, 2002).

Following their discovery, motor theories of action understanding proposed that mirror neurons might provide the basis for a matching mechanism between what we observe and what we can perform allowing the understanding of observed actions in motoric terms (Rizzolatti et al., 2001). Even if this hypothesis is strongly debated (Jacob and Jeannerod, 2005; Mahon and Caramazza, 2008; Hickok, 2009, 2013), a similar homologue mechanism has been proposed to exist in the human AON (Rizzolatti et al., 2001; Rizzolatti and Craighero, 2004; Rizzolatti and Sinigaglia, 2010).

In this brief overview, we will first describe previous fMRI studies that investigated how motor experience affects activation within the AON, and to which degree these studies allow drawing conclusions about the role of this network in action understanding. As the majority of the studies investigated only the AON and given the limited scope of this Opinion, we will focus on this network, even if our considerations might also hold true for other areas. We will then try to delineate how future studies might exploit motor expertise as a tool for gaining insights into the neural basis of action understanding.

## Recent neuroimaging findings on motor expertise in action observation

Following motor theories of action understanding, changes in motor repertoire should modify the brain response within the AON while observing these newly acquired actions. Starting from this assumption, most studies on expertise investigated how the acquisition of a skilled action, such as sport or dance moves, affects AON activity while observing the same movement.

Most of the contributions investigating motor expertise while observing sport actions are limited to one or few studies within the same domain, such as archery (Kim et al., 2011), badminton (Wright et al., 2010, 2011) or basketball (Abreu et al., 2012). Typically, these studies compare the blood-oxygen-level-dependent (BOLD) response between experts and novices. Although these studies considered different tasks and comparisons of experimental conditions, they seem to suggest a stronger activation for experts in comparison to novices not limited to the AON but recruiting also other brain regions (see Figure 1B). However, an interpretation of these results is difficult as, in addition to extensive practice of the observed movements, experts also have a strong visual familiarity with the observed stimuli which might affect the BOLD effect within the very same regions.

Beside these sparse investigations on different sport actions, a more systematic investigation involved the effect of dance expertise on activity within the AON (Calvo-Merino et al., 2005, 2006; Cross et al., 2006, 2009a,b, 2012, 2013; Pilgramm et al., 2010). Calvo-Merino et al. (2005) measured the BOLD effect of ballet dancers, capoeira dancers, and non-dancers watching two different types of dance movements (ballet or capoeira moves). They found a stronger recruitment of several regions within the AON (bilateral PMD, bilateral SPL and AIP, left PMV and left STS) in ballet and capoeira dancers for the observation of the trained in comparison to the untrained dance style, whereas they found no difference between the two dance styles in the non-dancers.

Calvo-Merino et al. argued that the activation for the trained in comparison to the untrained dance style was due to simulation of those actions that were within the motor repertoire of the dancer. Alternatively, as pointed out above, dancers' strong visual familiarity with the observed stimuli might affect the measured difference in BOLD effect.

In a follow up study, Calvo-Merino et al. (2006) investigated this issue by trying to disentangle the different contributions of visual familiarity and motor practice on the BOLD effect within the AON of expert dancers. They exploited the fact that some ballet movements are gender-specific while others are commonly performed by both male and female dancers. Calvo-Merino et al. (2006) found that activity within several regions of the AON (left PMD, bilateral AIP) was higher when observing actions within the observer's motor repertoire. However, visual familiarity might have played a role also in this study since dancers might have gathered more visual experience with those movements that are part of their own motor repertoire.

Another series of studies by Cross et al. (2006, 2009a,b) explored how activity related to action observation is modified after the acquisition of motor (physical practice) and/or visual experience (visual practice) with specific dance actions. These authors demonstrated stronger activity within AON regions during the observation and imagination of observed actions which were previously trained physically in comparison to actions that were not (Cross et al., 2006). In a subsequent study Cross et al. (2009a,b) showed that both previous physical and visual practice of dance sequences modulates activity within the AON while observing dance movements.

Figure 1B shows the peaks of activations for the different motor expertise studies. It is evident that there seems to be a consistent recruitment of premotor and parietal nodes of the AON for observing trained with respect to untrained moves, but, at the same time, there is also a widespread recruitment of other brain regions.

These studies suggest an effect of motor expertise on AON activation while *perceiving* an action, but it is difficult to assess the involvement of the AON in action *understanding* as none of these studies adopted a task directly investigating this process in a quantitative manner. Action understanding is intended here as the distinction between different actions irrespective of the properties (e.g., kinematics, goal, environmental cues, etc.) adopted to achieve such discrimination. We will elaborate on this point in the final section.

## Future perspective: using motor expertise to study action understanding

In this section, we discuss possible ways of testing the proposed role of the AON in action understanding. If the ability to understand actions depends on sensory-motor representations of these actions, then an experience-based modification (either impairment or improvement) of these representations should lead to a corresponding measurable modification in the ability to understand these actions, as in tasks involving action recognition. Crucially, it is also necessary to discount the possible role of regions outside this network (e.g., the *mentalizing system*).

Motor expertise might serve as an interesting tool to test the involvement of areas within and outside the AON in action understanding. However, one of the problems to overcome is making sure that the learned movements were not previously experienced by the participants. As most everyday actions are physically or visually experienced during normal development, the new acquisition of complex movements, such as sport and dance moves, allows to more easily control for possible confounds related to previous exposure or practice of the studied movements. Another problem to face is that performance might be close to ceiling in tasks using natural stimuli (videos or pictures of actions), making it difficult to find a modulation of performance as a function of motor experience. One possibility to overcome this issue could be to use point-light display (Johansson, 1973). This stimulation recruits part of the AON (Saygin et al., 2004; Wright et al., 2011), and its perception has been shown to be affected by motor expertise (Casile and Giese, 2006). Furthermore, the adoption of point-light display might mitigate visual familiarity confounds as they do not resemble a “natural” stimulation, and they can be easily manipulated in order to disrupt the perceived movement simply by adding noise. A recent study (Lingnau and Petris, 2013) adopted this approach and observed that the ability to understand actions decreased with increasing noise level.

This approach could be adopted to investigate differences in action understanding, using point-light display with different level of noise, within the same individual on trained and untrained stimuli after different types of practice (as in Cross et al., 2006, 2009a,b). In addition to the possible effects of visual and physical practice, a motor-only training could be introduced where physical practice might be performed blindfolded in order to eliminate potential visual confounds (as in Casile and Giese, 2006). These different types of training might affect common or different parts of the brain during action understanding. Crucially, motor learning without visual feedback alone could determine a modification in action understanding performance and a related functional modification within or outside regions of the AON. This could demonstrate that motor learning alone might have an effect on visual recognition of trained actions, avoiding interpretational confounds induced by a concomitant visual learning.

We have highlighted motor expertise as an interesting experimental manipulation to comprehend the role of the AON in action understanding. Further, these studies will profit strongly from the adoption of new MVPA decoding techniques (Kriegeskorte and Bandettini, 2007) as they allow a more fine-grained distinction (e.g., between different types of observed or executed actions, see also Oosterhof et al., 2013) that are not possible to reveal with univariate methods. This could be especially useful to assess decoding accuracy modifications between different actions (e.g., move A vs. move B) based on the type of training (trained vs. untrained) with different levels of noise. Further, changes in decoding accuracy between different actions before and after training might be also informative regarding the regions affected by the different types of training (physical, visual or motor-only).

To conclude, this Opinion focused on describing neuroimaging investigations on action perception/understanding, which are correlational in nature. It is not possible to define a causal link between such results and concomitant behavioral changes. However, these studies might provide interesting starting points for future studies using TMS in healthy participants or voxel-based lesion-symptom mapping in brain damaged patients.
